# Association between metabolic disorders and seminal plasma miRNA levels: a pilot study

**DOI:** 10.1186/s12610-022-00159-7

**Published:** 2022-06-07

**Authors:** Sarah Saget, Laurent Kappeler, Valérie Grandjean, Patricia Leneuve, Isabelle Berthaut, Céline Faure, Sébastien Czernichow, Chrystèle Racine, Rachel Lévy, Charlotte Dupont, Metasperme Collaboratif group, Nathalie Sermondade, Florence Eustache, Catherine Patrat, Myriam Benarroch, Isabelle Cedrin, Vanina de Larouzière, Emmanuelle Mathieu D’Argent, Angela Sutton, Jérôme Guechot

**Affiliations:** 1 Sorbonne Université, INSERM, Centre de Recherche St-Antoine, CRSA, 75012 Paris, France; 2 IHU-ICAN Institute of Cardiometabolism and Nutrition, Paris, France; 3 Inserm U1065, Team Control of Gene Expression (10), Université Cote d’Azur, Nice, France; 4 Service de Biologie de La Reproduction CECOS, Hôpital Tenon, AP-HP.Sorbonne-Université, 75020 Paris, France; 5 Service de Nutrition, Université de Paris, Hôpital Européen Georges Pompidou, AP-HP Paris, France

**Keywords:** Seminal plasma, MiRNA, Metabolic disorders, Metabolic syndrome, Anthropometric parameters

## Abstract

**Background:**

Excess weight and metabolic disorders have a negative impact on male reproductive functions. The mechanisms involved are numerous and complex and epigenetic mechanisms may also be involved, notably through the small non-coding RNAs. Among them, microRNAs (miRNAs) are of particular interest. This preliminary study aimed to identify the miRNAs differentially enriched in seminal plasma related to metabolic disorders and if some are also associated with spermatic parameters alterations. One hundred and sixty men between 18 to 45 years, partners of infertile couple, were included in this cohort. The miRNAs associated with metabolism were selected from the literature and assayed by quantitative real-time PCR using TaqMan gene expression assays. A subset of those with an interesting profile in seminal plasma were secondarily tested in blood.

**Results:**

Among the 11 selected miRNAs, seven were detected in seminal plasma (miR10b, miR19a, miR19b, miR34b, miR34c, miR133b, miRlet7c). A negative correlation was observed between seminal miR19a levels and metabolic syndrome, blood glucose and C-peptide. Seminal miR19b levels were also negatively correlated with metabolic syndrome. Seminal miR34c levels were negatively correlated with body mass index (BMI) and waist circumference. Seminal miR133b levels were positively correlated with BMI, waist circumference and leptin levels. Interestingly, modifications of miRNAs in seminal plasma seem specific since highlighted above correlations were not retrieved in the blood plasma for the miR19a, 19b, 10b, 34c.

**Conclusion:**

Few metabolic and anthropometric disorders are correlated with the level of specific miRNAs in seminal plasma. Further studies will be required to decipher if other small non-coding RNAs may also be correlated with metabolic and anthropometric disorders and to assess their potential implication in the alteration of reproductive functions in men with obesity or metabolic disorders.

**Clinical study:**

Metabolic Syndrome and Male Infertility (Metasperme): Trial registration: NCT01974947. Registered 18 July 2013.

## Résumé

**Contexte:**

L’excès de poids et les troubles métaboliques ont un impact négatif sur les fonctions de reproduction masculine. Les mécanismes impliqués sont nombreux et complexes, et des mécanismes épigénétiques peuvent également intervenir, notamment par le biais des petits ARN non codants. Parmi eux, les microRNAs (miRNAs) présentent un intérêt particulier. Cette étude préliminaire visait à identifier les miRNAs différentiellement enrichis dans le plasma séminal en relation avec des troubles métaboliques et si certains étaient également associés à des altérations des paramètres spermatiques. Cent soixante hommes âgés de 18 à 45 ans, partenaires de couple infertile, ont été inclus dans cette cohorte. Les miRNAs associés au métabolisme ont été sélectionnés dans la littérature et analysés par PCR quantitative en temps réel à l’aide de tests d’expression génique TaqMan. Un sous-ensemble de ceux présentant un profil intéressant dans le plasma séminal ont été secondairement testés dans le sang.

**Résultats:**

Parmi les 11 miRNAs sélectionnés, sept ont été détectés dans le plasma séminal (miR10b, miR19a, miR19b, miR34b, miR34c, miR133b, miRlet7c). Une corrélation négative a été observée entre les niveaux du miR19a séminal et le syndrome métabolique, la glycémie et le C-peptide. Les niveaux de miR19b séminaux étaient également corrélés négativement avec le syndrome métabolique. Les niveaux de miR34c séminaux étaient négativement corrélés avec l’IMC et le tour de taille. Les niveaux de miR133b séminaux étaient positivement corrélés avec l’IMC, le tour de taille et les niveaux de leptine. Il est intéressant de noter que les modifications des miRNA dans le plasma séminal semblent spécifiques puisque les corrélations mises en évidence ci-dessus n’ont pas été retrouvées dans le plasma sanguin pour les miR19a, 19b, 10b, 34c.

**Conclusion:**

Quelques désordres métaboliques et anthropométriques ont été observés corrélés avec le niveau de certains miRNAs dans le plasma séminal. Des études complémentaires sont nécessaires pour déterminer si d’autres petits ARN non codants sont corrélés aux troubles métaboliques et anthropométriques et pour évaluer leur implication potentielle dans l’altération des fonctions de reproduction chez les hommes souffrant d’obésité ou de troubles métaboliques.

## Background

Over the last few decades, couples have seemed to face increasing difficulties conceiving children. The decline of sperm parameters observed for half a century [1] may be involved. This reduction may be, in part, correlated with lifestyle and environmental disorders. Indeed, epidemiological studies have pointed out an increase of both obesity/overweight and metabolic disorders in the general population with an impact on male fertility. The first studies on the issue focusing on overweight and obesity observed an alteration of sperm parameters. Notably, a decrease in total sperm count [2] associated with a reduction in sperm cell quality, particularly DNA integrity [3]. More recently, researchers associated metabolic disorders and metabolic syndrome with deleterious impacts on male reproductive functions [4]. Among the numerous mechanisms possibly involved, hormonal dysfunction, chronic inflammation and systemic oxidative stress have been well-documented [5, 6]. Moreover, epigenetic modifications and small non-coding RNAs (sncRNAs) are increasingly evoked as potential additional mechanisms linking metabolic disorders and reproductive functions [7].

SncRNAs present with a size generally below 200 nucleotides and are not translated in functional proteins [8]. However, SncRNAs are involved in the post-transcriptional regulation of other gene expressions by affecting the stability and translation of messenger RNAs (mRNAs) [9]. SncRNAs involve micro-RNA (miRNA, miR), endogenous small interfering RNAs (endo-siRNAs), piwi-interacting RNAs (piRNAs) and transfer RNAs (tRNAs). Among sncRNAs, miRNAs present a particular interest and are the focus of most studies.

Recent studies also highlighted a crucial role of miRNAs in human fertility [10]. Indeed, regarding the reproductive function in males, miRNAs have been abundantly measured in both spermatozoa and seminal plasma [10, 11]. MiRNAs participate in gonadal development [12] and have been involved in gametogenesis [12]. Moreover, it has been reported that the sperm miRNA profile is modified along the journey through the epididymis [14]. Indeed, epididymosomes secreted by the epididymis play a critical role in the sperm maturation [15]. In addition, changes in miRNA profiles were also observed in testicular tissue, spermatozoa and seminal plasma in the case of sperm alteration [10]. For example, miR34b, miR34c and miR19a were observed to be down-regulated in testicular tissues, spermatozoa or seminal plasma of patients with oligozoospermia or azoospermia [16–19].

MiRNAs profiles can be modulated by environment and pathophysiological contexts, such as cancer, inflammation and metabolic disorders [20, 21]. Moreover, a recent review highlighted that oxidative stress generated by metabolic syndrome modifies the expression of many miRNAs involved in glucose and lipid metabolism regulation [22]. MiRNAs are also involved in the regulation of signalling pathways associated with inflammation, insulin sensitivity, and lipid metabolism [22]. Thus, miRNAs have emerged as key regulators of metabolic homoeostasis [9]. Interestingly some teams have observed an association between a high-fat diet and sperm miRNAs in rodents [23, 24], but to our knowledge, no association between metabolic disorders and miRNA profiles in semen has been established in humans.

The primary objective of the following study is to identify whether some miRNAs of potential interest were modulated in seminal plasma in the case of metabolic disorders, and if they were correlated with specific metabolic parameters. The secondary objective was to assess the potential association between seminal miRNA modulation and changes in sperm parameters. Finally, this work assessed whether the correlations observed for miRNAs in seminal plasma were independent or could also be retrieved in blood circulation.

## Materials and methods

### Patients

This study is an ancillary work of the national multicentric and transversal Metasperme study (Biomedical Research AOM 10,020 – NI – ID-RCB 2011–101,052-3), which focused on the evaluation of metabolic status and its relationship with sperm parameters in 160 male partners of infertile couples [6]. Men were recruited from July 2013 to January 2016 by a clinical practitioner (reproductive physician) or a biologist from the assisted reproductive technology department of one of three participating public centres in the Paris area.

Male eligibility criteria were (1) partner of a couple presenting primary or secondary infertility lasting longer than 12 months; (2) aged between 18 to 45 years; (3) insured with a social security scheme; (4) have had a prior medical consultation; (5) have completed informed and written consent to participate in the research study. Exclusion criteria were (1) problems understanding French; (2) smoking more than two packs a day; (3) monitored for viral risk; (4) male with an infertility that may be explained by identifiable factors in direct questioning (exposure to toxic products, infectious disease history, pathological anatomical background) or factors identified at the etiologic investigation at the sperm analysis (cytogenetic and genetic).

All patients in the Metasperme study signed a consent form for the analysis of epigenetic analyses to which the microRNAs belong. Ethical approval was obtained from the Ethics Committee of the University of Paris Ile-De-France. The Germethèque biobank (BB-0033–00,081) of Tenon’s hospital provided blood and semen samples and their associated data. Germethèque obtained consent from each patient to use their samples (CPP 2.15.27). The Germethèque pilotage committee approved the study design on 18 December 2015. The biobank has the required declaration #DC-2014–2202 and authorisation #AC-2015–2350. The present study request number made to Germethèque is #20,151,115, and its contract is referenced under the number CHUT: 18 313 C.

### Assessments

#### Anthropometric assessments

Height, weight (Tanita BC-420MA analyser) and waist circumference at the narrowest point between the lower border of the ribs and the iliac crest were evaluated. The body mass index (BMI) was calculated (kg.m^−2^). All patients were assessed by the same trained medical investigator using the same calibrated devices.

### Semen analyses

Semen samples were collected by masturbation in a sterile plastic cup after 3–5 days of sexual abstinence. After 30 to 60 min of semen liquefaction, conventional sperm parameters (volume, concentration and mobility) were evaluated according to WHO guidelines [25]. Sperm morphology was assessed on 200 spermatozoa using David’s criteria [26].

The remaining sperm sample was centrifuged (600G at room temperature for 10 min). The sperm pellet was used for the sperm DNA fragmentation assay and seminal samples were aliquoted and stored at − 80 °C for further miRNA analysis [3]. Sperm DNA fragmentation was detected with the terminal uridine nick end labelling (TUNEL) technique using an In Situ Cell Death Detection Kit, according to previously published methods (In Situ Cell Death Detection Kit, Fluorescein, Roche Applied Science) [3]. At least 200 spermatozoa were assessed, and the total DNA fragmentation rate was calculated as the number of positive cells divided by the total number of sperm nuclei.

### Blood samples and analyses

Blood samples were collected after a 12-h fasting period in dry tubes. After centrifugation 1900G at 4 °C for 10 min), the blood plasma was isolated and High-density lipoprotein (HDL) cholesterol, low-density lipoprotein (LDL) cholesterol, triglycerides and glucose concentrations were instantly measured through the standardised protocols of the hospitals’ biology laboratories. The remaining blood plasma was stored at − 80 °C for miRNA further analysis.

### Blood pressure assessment

Systolic and diastolic blood pressures were measured using a sphygmomanometer cuff around the patient’s arm after 5 min of bed rest in a supine position.

### Metabolic syndrome definition

Metabolic syndrome was diagnosed by the presence of at least three of the following criteria: waist circumference of more than 92 cm, triglycerides of 150 mg/dL/1.7 mmol/L or more, HDL cholesterol of less than 40 mg/dL / 1.0 mmol/L, fasting glucose of 100 mg/dL / 5.6 mmol/L or more, and arterial blood pressure of 130/85 mmHg or higher [27]*.*

### Measurements of miRNA levels

MiRNA from 20 µL of seminal plasma and from 300 µL of blood plasma were extracted from 160 patients using of the nucleospin miRNA plasma kit (Macherey–Nagel, Hoerdt, France) according to manufacturer instructions. The volume of seminal plasma used was adapted in view of its high concentration in miRNAs and contains a similar amount to those from the 300 µL of blood plasma [28]. Extra-diluent volume from the extraction kit was used for the dilution. Then, 2 µL of the extracted miRNA solution were linked with universal adapters and reverse transcribed using the TaqMan advanced miRNA cDNA synthesis kit (Thermo-Fisher Scientific, Wilmington, DE, USA) according to the manufacturer instructions. Quantitative real-time polymerase chain reaction (PCR) were performed in duplicate using validated TaqMan gene expression assays (Thermo-Fisher Scientific) and an ABI StepOnePlus PCR system (Thermo-Fisher Scientific).

A list of 11 miRNAs to be determined was chosen from the literature according to their associations reported in the field of male infertility (miR19a, miR19b, miR34b, miR34c, miR122) [10, 17–19, 29, 30] and/or metabolic alterations (miR19b, miRlet7c, miR133, miR10a, miR10b, miR183, miR340) [23, 24, 31–33]. Levels of these miRNAs were first determined in seminal plasma and four miRNAs (miR10a, miR122, miR183, miR340) were undistinguishable or to a lower concentration to be accurate. Then, the level of four miRNAs (miR19a, miR19b, miR34c, miR10b) highlighted in seminal plasma were then also determined in blood plasma. The abundance of each miRNA was measured against a standard curve added in each PCR plate to control the variation between each PCRs plate. The four points in duplicate of the standard curve were generated by the successive 5-time dilution of a pool mixed from seminal or blood plasma reverse-transcribed products of 50 individuals of the cohort and an association with arbitrary values following the dilution fold (i.e., 1000, 200, 40, 8). Next, each identified miRNA was normalised against the endogenous miR195-5p, which was also measured in each individual sample according the same method and accepted as stable in plasma [24].

#### Statistical analyses

A D’Agostino–Pearson normality test was used to assess the normality of the distribution. However, since most of the variables did not pass the normality test (alpha > 0.05), we used a nonparametric test. To compare men with metabolic syndrome and men without metabolic syndrome, we used a Fisher’s exact test for qualitative variables and a nonparametric Mann–Whitney test for quantitative variables. To measure the linear relationship between miRNA expression and metabolic/fertility parameters, we used Spearman’s correlation coefficient. Strength of correlation was calculated (r) and a *P* value < 0.05 was considered statistically significant. All statistical analyses were performed with Prism 7 for Mac OS X software (GraphPad software, Inc.).

## Results

### Patients characteristics

Of the 160 male partners of infertile couples in the Metasperme cohort, 47 presented with metabolic syndrome according to its definition (see Methods section). These subjects notably exhibited a higher BMI, waist circumference, systolic and diastolic blood pressure, triglycerides, and lower HDL cholesterol. In this cohort, some men presented with normal semen parameters while others did not. However, semen parameters in this subgroup of the Metasperme cohort do not reveal any statistically significant differences with those of the Metasperme cohort without metabolic syndrome (Table 1).

**Table 1:** Anthropometric. metabolic and spermatic characteristics of patients included in the study. Patients were allocated in two groups: the metabolic syndrome group (with MS) and the non-metabolic syndrome (no MS) group. *P* value corresponds to the difference between the metabolic and non-metabolic syndrome groups. Qualitative variables were analyzed using chi-square test. Quantitative variables were analyzed using the nonparametric Mann–Whitney test

	**All subjects (*n* = 160)**	**No MetS (*n* = 113)**	**MetS (*n* = 47)**	***P* value without vs with MetS**
Age (year)	37.1 ± 0.4	32.2 ± 0.4	39.1 ± 0.4	< 0.001
BMI (kg.m.^−2^)	26.4 ± 0.3	25.2 ± 1.1	29.3 ± 0.6	< 0.0001
Waist Circumference (cm)	92.9 ± 0.9	89.3 ± 1.0	101.3 ± 1.4	< 0.0001
Systolic blood pressure (mmHg)	125.6 ± 1.0	123.4 ± 0.9	130.7 ± 2	< 0.001
Glycaemia (mmol/l)	5.3 ± 0.1	5.1 ± 0.1	5.9 ± 0.2	< 0.0001
Total cholesterol (mmol/l)	5.2 ± 0.1	5.1 ± 0.1	5.4 ± 0.2	0.05
LDL cholesterol (mmol/l)	3.28 ± 0.07	3.23 ± 0.09	3.41 ± 0.14	0.18
HDL cholesterol (mmol/l)	1.23 ± 0.03	1.32 ± 0.03	1.02 ± 0.04	< 0.0001
Triglycerides (mmol/l)	1.7 ± 0.1	1.3 ± 0.1	2.5 ± 0.2	< 0.0001
Ejaculate volume (ml)	3.6 ± 0.1	3.6 ± 0.2	3.4 ± 0.2	0.49
Sperm concentration (10.^6^/ml)	50.3 ± 3.8	47.1 ± 4.3	59.2 ± 7.8	0.09
Total sperm count (10.^6^/ejaculate)	179 ± 17	173 ± 22	192 ± 29	0.16
Progressive motility (%)	39.9 ± 1.4	40.1 ± 1.6	39.5 ± 2.7	0.89
Sperm vitality (%)	71.6 ± 1.3	72.0 ± 1.5	70.7 ± 2.5	0.72
Sperm morphology (%)	16.0 ± 1.1	15.5 ± 1.4	15.3 ± 2.0	0.58
Sperm DNA fragmentation (%)	8.4 ± 0.5	8.3 ± 0.6	8.7 ± 0.8	0.33

*CRP* C-reactive protein, *DBP* Diastolic blood pressure, *FSH* Follicle-stimulating hormone, *LH* Luteinizing hormone, *SBP* Systolic blood pressure, *WC* Waist circumference

### miRNA dosages in seminal plasma and blood

From the 11 miRNAs selected, four were poorly or not detected (miR10a, miR122, miR183, miR340).

**Fig. 1:**
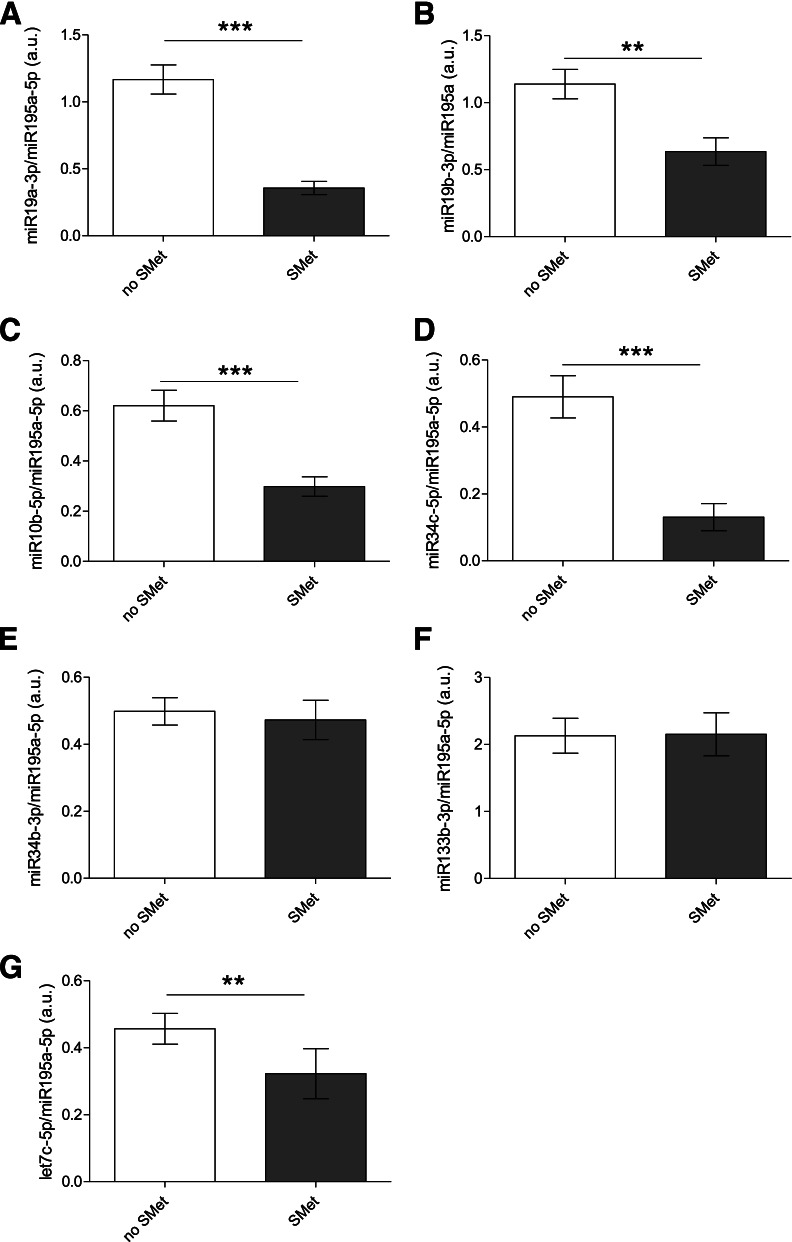
Relative miRNAs levels in patients with or without metabolic syndrome. Among the eleven miRNAs tested, seven miRNAs have been detected in seminal plasma by RT-qPCR, normalized against the miR195a-5p as endogenous control: **A** miR19a; **B** miR19b; **C** miR10b; **D** miR34c; **E** mir34b; **F** miR133b; **G** let7c. Comparisons of miRNAs levels between men with no metabolic syndrome (noSMet) and with metabolic syndrome (SMet) indicate 5 miRNAs with statistical difference with a nonparametric Mann–Whitney test, with *, *p* < 0.05; **, *p* < 0.01; ***, *p* < 0.001

Among the seven miRNAs detected in the seminal plasma (miR10b, miR19a, miR19b, miR34b, miR34c, miR133b, miRlet7c), five presented decreased levels in men with metabolic syndrome (Fig. 1). We did not observe any difference in miR195a-5 expression between the two groups. In addition, four exhibited statistically significative correlations with anthropometric or metabolic parameters (Table 2).". Notably, the miR19a, miR19b and miR34c levels were negatively associated with metabolic or anthropometric parameter alterations, while the miR133b level was positively correlated with anthropometric or metabolic parameters alterations (Table 2). More precisely, the level of miR19a was decreased in seminal plasma in the case of metabolic syndrome, and also presented a negative correlation with glycaemia (*r* =  − 0.178, *p* = 0.046) and C-peptide levels (*r* =  − 0.220, *p* = 0.015). MiR19a showed a tendency to negatively correlate with the other identified parameters but without reaching statistical significance. The seminal miR19b level was also decreased in seminal plasma in the case of metabolic syndrome. However, the miR19b did not present any statistically significant correlation when directly compared with identified metabolic and anthropometric parameters (e.g., *p* = 0.051 with waist circumference and *p* = 0.056 with triglyceride levels). The level of miR34c in seminal plasma was negatively correlated only with anthropometric parameters, namely BMI (*r* =  − 0.200, *p* = 0.040) and waist circumference (*r* = 0.258, *p* = 0.004). In contrast, the level of miR133b in seminal plasma was positively correlated only with anthropometric parameters, that is, BMI (*r* = 0.241, *p* = 0.015) and waist circumference (*r* = 0.275, *p* = 0.005). No statistically significant correlations were observed between seminal miR34b, miRlet7c or miR10b levels and anthropometric or metabolic parameters.

**Table 2:** Correlation between seminal miRNA and anthropometric and metabolic parameters

Anthropometric and metabolic parameters	miR 19a seminal	miR 19b seminal	miR 34b seminal	miR 34c	miR 133b	miR 10b	miR let-7c
Metabolic syndrome r p	**-0.304** ** < 0.001**	**-0.243** **0.004**	-0.138 NS	-0.151 0.096	-0.077 NS	0.012 NS	-0.101 NS
BMI r p	-0.155 0,089	-0,153 0.075	-0,085 NS	**-0.200** **0.040**	**0.241** **0.015**	0.030 NS	-0.068 NS
Waist circumference r p	-0.158 0.079	-0.166 0.051	-0.149 0.084	**-0.258** **0.004**	**0.275** **0.005**	-0.032 NS	-0.104 NS
Glycaemia r p	**-0.178** **0.046**	-0.131 NS (0.12)	-0.078 NS	-0.098 NS	0.072 NS	-0.004 NS	-0.134 NS
Total cholesterol r p	-0.039 NS	-0.113 NS	-0.035 NS	-0.033 NS	-0.041 NS	-0.046 NS	> -0.001 NS
LDL cholesterol r p	-0.031 NS	-0.081 NS	-0.037 NS	-0.025 NS	-0.031 NS	-0.002 NS	-0.021 NS
HDL cholesterol r p	-0.154 0.079	-0.086 NS	-0.139 NS	-0.074 NS	-0.078 NS	-0.097 NS	-0.079 NS
Triglycerides r p	-0.157 0.079	-0.161 0.056	-0.109 NS	-0.096 NS	-0.067 NS	-0.024 NS	-0.111 NS
Insuline r p	-0.154 0.09	-0.083 NS	-0.069 NS	-0.108 NS	-0.022 NS	0.135 NS	-0.057 NS
C-peptide r p	**-0.220** **0.015**	-0.100 NS	-0.091 NS	-0.114 NS	-0.090 NS	0.129 NS	-0.131 NS

To make reading the table easier, the significant values are indicated in bold characters

*BMI* Body mass index

*r* Pearson coefficient correlation, *p* < 0,05: significant

Regarding semen parameters, the level of miR34b in seminal plasma was significantly negatively correlated with sperm DNA fragmentation (*r* =  − 0.185, *p* = 0.047). No other correlation between seminal miRNAs and conventional semen parameters was observed.

Four miRNAs (i.e., miR10b, miR19a, miR19b, miR34c) were selected and their analysis was extended to blood plasma. As expected, all four of the miRNAs were detected in blood plasma. However, levels of miR19a, miR19b and miR10b presented a different profile in blood plasma than in seminal plasma. Indeed, the level of miR19a in blood plasma was negatively correlated with total cholesterol (*r* =  − 0.16, *p* = 0.03; Table 3). This modification was not strong enough to be associated with a significant decrease in males with metabolic syndrome (data not shown). Similarly, the level of miR19b in blood plasma was negatively correlated with total- (*r* =  − 0.19, *p* = 0.01) and LDL- cholesterol (*r* =  − 0.18, *p* = 0.02). However, levels of miR34c and miR10b in blood plasma were not significantly correlated with neither anthropometric nor metabolic status. Interestingly, no correlation was observed between the blood miRNA and semen miRNA levels (data not shown).

**Table 3:** Correlation between blood miRNA and anthropometric and metabolic parameters

Anthropometric and metabolic parameters	miR 19a blood	miR 19b blood	miR 34c	miR 10b
Total cholesterol r p	**-0.16** **0.03**	**-0.19** **0.01**	-0.18 NS	-0.005 NS
LDL cholesterol r p	-0.16 NS	**-0.18** **0.02**	-0.10 NS	-0.04 NS

To make reading the table easier, the significant values are indicated in bold characters

Other anthropometric and metabolic parameters were tested at the same time, but were not significant, therefore not added to the table

*r* Pearson coefficient correlation, *p* < 0,05: significant

## Discussion

Obesity and metabolic disorders have been described as risk factors for male infertility and the alteration of semen parameters, such as sperm concentration and sperm DNA fragmentation [2–4]. The underlying mechanisms are multiple and complex. Hormonal imbalances, inflammation and oxidative stress are the most commonly evoked instigators. However, recent hypotheses refer to epigenetic changes associated with overweight and obesity that may also directly alter sperm quality. Accordingly, data from animal models have shown the impact of a high-fat diet on DNA methylation [31] or on miRNA profiles in murine sperm cells [24]. MiRNAs are involved in reproductive functions and are abundant in spermatozoa and seminal plasma [10]. Some miRNAs present in testes, spermatozoa and seminal plasma have been found to be downregulated (miR34b, miR34c, and miR19a, miR122) or upregulated (miR429) in the case of sperm alterations while the underlying mechanisms remain unknown [16–19, 29, 30].

To our knowledge, we have shown for the first time that some miRNAs in seminal plasma can be associated with male metabolic or anthropometric profiles. Interestingly, blood glucose, weight (BMI) and abdominal obesity (weight circumference) correlated with certain miRNAs in seminal plasma while the lipid profile did not. Although few studies have shown that dyslipidemia can have repercussions on male reproductive functions [34], an increasing number of studies present the increased glycaemia and abdominal obesity as risk factors for male infertility [4, 35]. Unlike other teams [10], we did not find large correlations between the miRNAs studied and conventional sperm parameters although we observed that the levels of miR34b was negatively correlated with sperm DNA fragmentation. Sperm DNA alteration may be associated with impaired fertilisation and embryo development [36], and it is now accepted that sperm quality, assessed by DNA integrity, may be challenged by metabolic disorders and obesity [3, 37]

In this study, seminal miR34b, miR34c, miR133, miR19a, miR19b seminal miRNAs were observed to be modified by metabolic disorders.

MiR34 family members are located on two separate chromosomal loci (MiR34a and MiR34b/c) [38]. They have been well-characterised as a tumour suppressor [39]. However, they also play a critical role in non-cancerous diseases. Indeed, they have been reported to have function in cardiovascular disorders by regulating apoptosis, telomere attrition, DNA damage, and inflammatory response [40]. Interestingly, they are also involved in male reproductive functions since both miR34b and miR34c have been observed up-regulated during murine postnatal testicular development and spermatogenesis [39, 40].

MiR133b has been observed enriched in muscle and heart and in brown adipose tissue. It plays a critical role in pathological cardiac hypertrophy, but also in metabolic homeostasis [41]. It is an inhibitor of brown adipocyte differentiation and is involved in energy balance [42]. Concerning reproductive functions, miR133b is up-regulated in Sertoli cells in men with Sertoli-Cell-Only syndrome. Consequently, it may play a role in spermatogenesis since Sertoli cells regulate spermatogenesis [43]. In this study, we observed that mir133b levels were increased in case of metabolic disorders although it was not associated with sperm parameters alterations.

MiR19a and miR19b belong to the miR19 family and arise from 2 clusters miR17-92 and miR106a-363 [44]. They are best known in the field of oncology, but they have also been described in context of cardiac, vascular or neurological disease [45]. They were also observed as dysregulated in case of male infertility [10] and may be involved in male reproductive functions as well as playing a role in offspring programming. Indeed, miR19a and miR19b were fund dysregulated in testis and sperm of mice submitted to western diet [23]. When miR19b was injected into one-cell embryo of wild type mice, offspring have developed the same metabolic alterations as the offspring of male rats fed the western diet [23]. In the present study, in humans, we observed a negative association between miR19a and miR19b levels and metabolic or anthropometric disorders. This may illustrate a potential inter-generational adaptation underlying mechanism in Human.

In blood, we confirm that the miR19a and miR19b were associated to the men metabolic status [9, 22]. Interestingly, contrary to what is described in the literature [46], we did not observe any correlation between miR10b and metabolic parameters, Moreover, no association between seminal plasma miRNAs and blood plasma miRNAs levels was highlighted. This is of importance as it may suggest that miRNA levels in seminal plasma may be differentially regulated. Many questions remain, including the mechanisms by which miRNAs arrive in seminal plasma and its regulation. It seems unlikely to be through only passive diffusion since there is no clear correlation between seminal and blood miRNA. Small extracellular vesicles (sEVs) of seminal plasma are secreted by epididymis and contain miRNAs that are sensitive to physiological conditions and could be a source. The epididymis plays a crucial role in secretion and excretion of epididymosomes which differs from region to region [47, 48]. Moreover, the seminal vesicles and the prostate, which produce almost the whole seminal plasma, could also play a critical role in seminal plasma miRNAs profile [49]. Further studies would be necessary to understand if these miRNAs impact sperm quality and fertility [49].

In addition to sperm development and maturation, miR19a, miR19b, miR34c and miR133b, that were associated to metabolic disorders, may also be directly involved in fertilisation and contribute to embryo development. MiR34c appear crucial for the egg first divisions [50–52]. Moreover, miRNAs may also play a role in paternal transmission of non-communicable diseases [53]. Thus, it was observed that direct injection of miR-19b in normal healthy zygotes induced metabolic disorders in offspring [23]. The concept of paternal origins of health and disease (POHAD) [54] emerged several years ago. POHAD maintains that the paternal environment at conception may have an impact on the development of offspring and long-term health. To explain this programming, the role of sperm has been widely studied, including epigenetic changes in sperm cells, such as DNA methylation, histone modification and miRNA profiles [55–57]. However, seminal plasma is rarely investigated in this context. Nevertheless, seminal plasma interacts with the female genital tract and may influence pregnancy onset. Additionally, seminal plasma triggers the process of immune adaptation by the mother [58]. The mechanisms are probably more complex since the seminal plasma contains many nutrients, proteins, molecules and genetic material (miRNA) that will be involved in uterine remodelling, embryo implantation and foetal development [59].

More importantly, epididymosomes are able to convey protein cargo to the sperm [47] that can then be integrated in the zygote. For example, it was observed that the miRNAs present in the epididymis cauda are regulated by glucocorticoid receptors and are involved in the transmission of paternal stress programming to offspring [60]. If metabolic and anthropometric disorders influence miRNAs profile in seminal plasma, and if these miRNAs are incorporated in sperm and then in zygotes, this transmission pathway should be considered as a possible mechanism for the impact of an inadequate paternal environment on children’s health. Further studies are required to confirm these hypotheses.

### Limitations

In this preliminary study, we tested only 11 miRNAs to validate our hypothesis. These first results are promising and highlight the need to apply a high throughput approach, such as miRSeq, to identify miRNAs that are potentially involved. Furthermore, this study only involved male partners of infertile couples. It might be interesting to conduct a similar study on another population with different subgroups of males, regarding their metabolic status and sperm parameters.

## Conclusion

This study showed that metabolic or anthropometric disorders may be correlated with the expression of certain miRNAs in seminal plasma. Only a few miRNAs could be tested, but this pioneering study shows the usefulness of quantifying seminal sncRNAs to evaluate the impact of metabolic disorders on metabolic changes in seminal plasma. Further studies are needed to decipher whether sncRNAs are involved in reproductive function alteration in obese men with or without metabolic disorders. In addition to this impact, these deregulated miRNAs could play a role in the paternal transmission of developmental diseases (POHAD).

## Data Availability

Data are the property of the Public Assistance – Paris Hospitals [Assistance Publique – Hôpitaux de Paris (AP-HP)] that does not authorise as a promoter the sharing of data without a contract. Consultation by the editorial board or interested researchers may nevertheless be considered.

## Abbreviations

BMI: Body mass index
endo-siRNAs: Endogenous small interfering RNAs
miRNA: Micro-RNA
piRNAs: Piwi-interacting RNAs
sncRNAs: Small non-coding RNAs
tRNAs: Transfer RNAs
